# Hepatoprotective Effect of Carboxymethyl Pachyman in Fluorouracil-Treated CT26-Bearing Mice

**DOI:** 10.3390/molecules22050756

**Published:** 2017-05-06

**Authors:** Canhong Wang, Xiaowei Huo, Li Gao, Guibo Sun, Li Cao

**Affiliations:** 1 Institute of Medicinal Plant Development, Chinese Academy of Medical Sciences and Peking Union Medical College, Beijing 100193, China; canhongwang@126.com (C.W.); huoxiaoweiforever@163.com (X.H.); ligaoimplad@163.com (L.G.); guibosun1@163.com (G.S.); 2 Hainan Branch Institute of Medicinal Plant Development, Chinese Academy of Medical Sciences and Peking Union Medical College, Haikou 570311, China

**Keywords:** carboxymethyl pachyman, fluorouracil, hepatoprotective effect, antiinflammation, antioxidant, antiapoptosis

## Abstract

5-Fluorouracil (5-FU) is the chemotherapeutic agent of first choice for the treatment ofcolorectal cancer, however, treatment-related liver toxicity remains a major concern. Thereby, it is desirable to search for novel therapeutic approaches that can effectively enhance curative effects and reduce the toxic side effects of 5-FU. Carboxymethyl Pachyman (CMP) exhibits strong antitumor properties, but the antitumor and hepatoprotective effects of CMP and the molecular mechanisms behind these activities, are however poorly explored. Thereby, the purpose of the present study was to evaluate the hepatoprotective effect of CMP in 5-FU-treated CT26-bearing mice, and further explore the underlying mechanism(s) of action. Initially, a CT26 colon carcinoma xenograft mice model was established. The immune organ indexes, blood indicators, liver tissue injury, and indicators associated with inflammation, antioxidant and apoptosis were then measured. Our results showed that CMP administration increased the tumor inhibitory rates of 5-FU and, meanwhile, it reversed reduction of peripheral white blood cells (WBC) and bone marrow nucleated cells (BMNC), increase of alanine aminotransferase (ALT) and aspartate aminotransferase (AST), and decrease of superoxide dismutase (SOD), catalase (CAT), GSH-Px and glutathione(GSH) induced by 5-FU. Moreover, CMP in combination with 5-FU alleviated severe liver injury induced by 5-FU via reducing the levels of ROS, IL-1β, and IL-6, decreasing expression of p-IκB-α, NF-κB, p-NF-κB, pp38 and Bax, and elevating levels of Nrf2, GCL, HO-1 and Bcl-2. Collectively, these outcomes suggested that CMP effectively enhanced the curative effects of 5-FU and simultaneously reduced the liver injuries induced by 5-FU in CT26-bearing mice, and the mechanism may be associated with regulation of NF-κB, Nrf2-ARE and MAPK/P38/JNK pathways.

## 1. Introduction

Colorectal cancer, accompanied by intestinal microflora disorders, inflammation, metabolic dysfunction symptoms, and invasion of pathogens, remains the third most commonly diagnosed cancer and the fourth leading cause of cancer-related mortality worldwide [1,2,3]. Besides surgery, chemotherapy appears to be a primary therapeutic strategy for colorectal cancer largely due to the fact that most patients present at diagnosis with unresectable or metastatic disease [4].

5-Fluorouracil, a pyrimidine analogue that interferes with thymidylate synthesis, is a basic chemotherapeutic agent for the treatment of colorectal cancer [5,6], however, the development of drug-resistant phenotypes and systemic toxicity, especially liver injury, have become severe limiting factors in 5-FU therapy [7,8,9], and thereby 5-FU alone is of limited benefits in enhancing the survival of patients with advanced colorectal cancer. Emerging evidence indicates that 5-FU-induced liver injury is associated with multiple mechanisms, including 5-FU-induced inflammation, oxidative stress, and hepatocyte apoptosis [10,11]. Hence, the development of ideal strategies that could enhance therapeutic effectiveness of 5-FU, simultaneously reduce its toxicity to liver via regulating inflammation, oxidative stress, and hepatocyte apoptosis, without compromising its tumoricidal action is highly warranted.

Recently, suitable drug combinations, such as the combination of 5-FU, leucovorin calcium, oxaliplatin, and irinotecan, were believed to enhance the synergy, and the interactions of chemical constituents within the combination are thought to be responsible for the improvement of therapeutic efficacy over 5-FU treatment alone [12,13]. While the anti-cancer effect of this regimen is promising, therapy-associated toxicity remains a concern limiting its clinical use. Traditional Chinese medicines have been used for a long history to enhance physical performance [14], and in many cases natural products and their derivatives are thought to be therapeutically superior to chemically synthesized drugs [15], such as docetaxel and topotecan approved by FDA, which have been demonstrated clinical utility based on the ongoing investigations [16]. Therefore, natural products or their combinations with currently approved chemotherapeutic drugs are well accepted as alternative remedies in anticancer therapy, especially in countries where herbal medicines are widely used [17].

*Poria cocos* (Chinese name: Fu Ling), the dried sclerotia of *P. cocos* (Schw.) Wolf(Polyporaceae), has drawn increasing attention as an important traditional medicine and nutrition food in China and other Asian countries [18,19], because it is generally plentiful and considered relatively non-toxic in clinical practice. Pachyman, with water-insoluble (1→3)-β-d-glucan as its main constituent [20], exerts mild antitumor activity, however, its carboxymethylated derivative, carboxymethylated (1→3)-β-d-glucan (CMP), exhibits improved antitumor activity [21,22].Recently CMP has been extensively investigated to explore its potential anti-tumor activity in various cancers and the underlying mechanism was revealed to be associated with the enhancement of immune response [22], indicating that CMP may help protect against 5-FU-induced liver injury as well as potentiate the antitumor effects of 5-FU. However, presently, its hepatoprotective effect and its use as an effective sensitizer for 5-FU therapy, as well as the underlying mechanism of the beneficial effects are yet poorly known.

We, therefore, aimed to investigate the synergistic and hepatoprotective effect of CMP in a 5-FU-treated CT-26 xenograft mice model, and further elucidate the underlying mechanisms of these actions. The results indicated that the combined therapy with CMP and 5-FU potentiated the inhibitory effect of 5-FU on CT-26 xenografts, meanwhile, it alleviated the liver injury caused by 5-FU, via regulating NF-κB, Nrf2-ARE and MAPK/P38/JNK pathways. Collectively, CMP may be a promising and beneficial agent for use in combination with 5-FU for the improvement of clinical chemotherapy results.

## 2. Results

### 2.1. Effect of the Combination with CMP and 5-FU on Tumor Inhibition Ratio, Body Weight, Organ Indexes, WBC and BMNC of CT26-Bearing Mice

**Figure 1:**
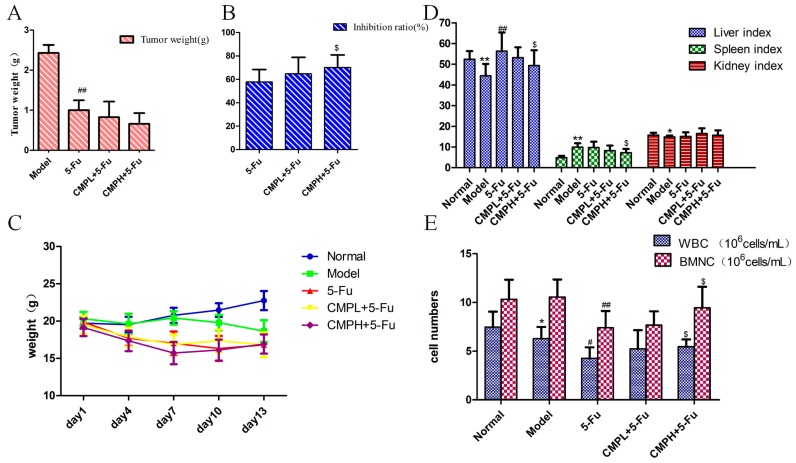
The effect of the combination treatment with CMP and 5-FU on tumor inhibition. (**A**,**B**) Tumor weight and tumor inhibition ratio of the combination treatment. Data are presented as mean ± SD. ^##^ *p <* 0.01 vs. Model group; ^$^ *p <* 0.05 vs. 5-FU group. (**C**) Mice weight during drug administration. (**D**) Effect of the combination treatment on organ indexes. Organ indexes were calculated by the following formula: Organ index = organs weight (mg)/body weight (g). Data are presented as mean ± SD. * *p <* 0.05, ** *p <* 0.01 vs. Normal group; ^##^ *p <* 0.01 vs. Model group; ^$^ *p <* 0.05 vs. 5-FU group. (**E**) Effect of the combination treatment on WBC and BMNC. Data are presented as mean ± SD. * *p <* 0.05 vs. Normal group; ^#^ *p <* 0.05, ^##^ *p <* 0.01 vs. Model group; ^$^ *p <* 0.05 vs. 5-FU group.

5-FU significantly inhibited CT26 carcinoma growth as indicated by decreased tumor weight when compared with the model group (*p* < 0.01) (Figure 1A). Moreover, CMPH (100 mg/kg) combined with 5-FU substantially increased tumor inhibitory activity, incomparison with the administrations of 5-FU alone (*p* < 0.01), with no significant effect on body weight (Figure 1B,C). The spleen index could reflect the immune function of the organism. The results in Figure 1D show that 5-FU treatment significantly increased the spleen index when compared with the untreated mice, which was then obviously reduced by combination with CMPH and 5-FU. The increased liver index induced by 5-FU was also significantly alleviated by the combination treatment with CMPH and 5-FU. Both 5-FU and the combined treatment had no effect on kidney index. Additionally, the results of the hematological analyses (Figure 1E) showed that, the numbers of WBC and BMNC in 5-FU group were significantly decreased compared with that in the model group. The combination with CMPH and 5-FU, however, obviously reversed the decrease of WBC and BNMC, indicating the improvement of the immunity.

### 2.2. Effect of the Combination with CMP and 5-FU on Liver Injury

**Figure 2:**
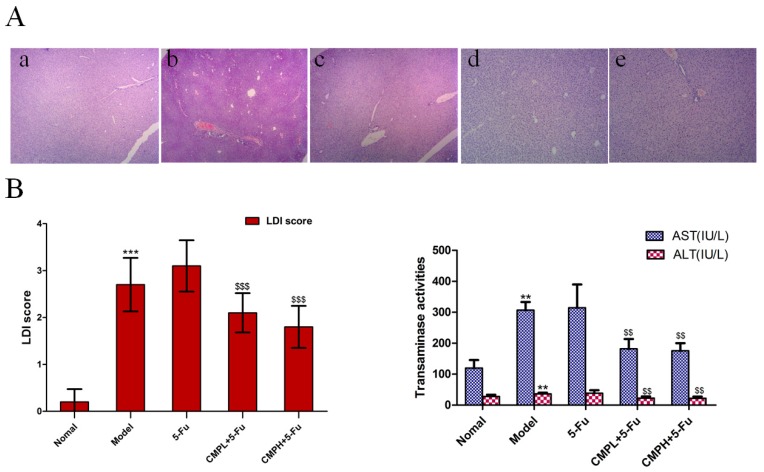
Hepatoprotective effect of the combination treatment with CMP and 5-FU. (**A**) Hematoxylin and eosin (H&E) staining of livers. Mice were treated with saline, 5-FU (25 mg/kg), 5-FU (25 mg/kg) + CMP (50 mg/kg, CMPL), and 5-FU (25 mg/kg) + CMP (100 mg/kg, CMPH), for 14 days, mice were then sacrificed and livers were excised and stained with hematoxylin and eosin (magnification 400 ×). Groups represent, **a**: normal mice + saline; **b**: model mice + saline; **c**: model mice + 5-FU 25 mg/kg; **d**: model mice + 5-FU 25 mg/kg + CMP 50 mg/kg; **e**: model mice + 5-FU 25 mg/kg + CMP 100 mg/kg. (**B**) LDI score to evaluate histological damage of liver tissues: 0, absent (no demolishment); 0.5, few (0%–20% demolishment); 1, mild (20%–50% demolishment); 2, moderate (50%–80% demolishment); 3, severe (80%–100% demolishment). Data are presented as mean ± SD. *** *p <* 0.001 vs. Normal group; ^$$$^ *p <* 0.001 vs. 5-FU group. (**C**) Serum alanine aminotransferase (ALT) and aspartate aminotransferase (AST) levels. ALT and AST levels in serum were assayed using a commercially available test kits according to the manufacturer’s instructions. Data are presented as mean ± SD. ** *p <* 0.01 vs. Normal group; ^$$^ *p <* 0.01 vs. 5-FU group.

After two weeks of treatment, the livers were collected and embedded in paraffin for HE analysis. As shown in Figure 2, severe injury occurred in liver tissues of the mice bearing CT26 xenografts, which was further aggravated by 5-FU treatment, as shown by marked neutrophil infiltration and necrosis in the liver tissues, significantly elevated LDI sore, and dramatically increased serum levels of ALT and AST. Fortunately, the combined treatment strongly reduced liver tissue injury, and levels of ALT and AST were also significantly decreased in CMP plus 5-FU group in comparison with 5-FU treatment alone. These results supported the idea that CMP could significantly relieve liver injury caused by 5-FU treatment.

### 2.3. Antioxidative Effect of the Combination with CMP and 5-FU on Liver Tissues

**Figure 3:**
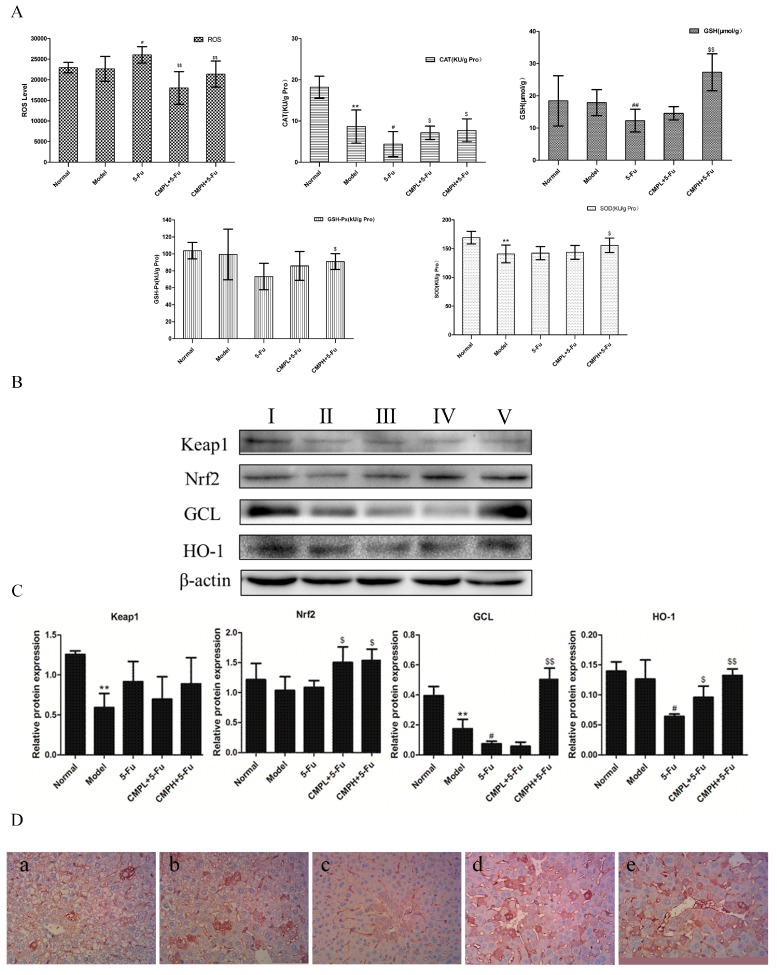
Antioxidant effect of the combination treatment with CMP and 5-FU on liver tissues. (**A**) The effect of the combination treatment with CMP and 5-FU on levels of ROS, SOD, GSH, GSH-Px, and CAT in liver tissues. Data are presented as mean ± SD. ** *p <* 0.01 vs. Normal group; ^#^ *p <* 0.05, ^##^ *p <* 0.01 vs. Model group; ^$^ *p <* 0.05, ^$$^ *p <* 0.01 vs. 5-FU group. (**B**) Liver tissues were isolated to analyze the expression of Nrf2, Keap1, HO-1, and GCL by western blot analyses. Groups represent, **І**: normal mice + saline; **ІІ**: model mice + saline; **ІІІ**: model mice + 5-FU 25 mg/kg; **ІV**: model mice + 5-FU 25 mg/kg + CMP 50 mg/kg; **V**: model mice + 5-FU 25 mg/kg + CMP 100 mg/kg. (**C**) Semi-quantitative analysis of western blot. Data are presented as mean ± SD. ** *p <* 0.01 vs. Normal group; ^#^ *p <* 0.05 vs. Model group; ^$^ *p <* 0.05, ^$$^ *p <* 0.01 vs. 5-FU group. (**D**) Immunohistochemical staining of Nrf2 in liver tissues (magnification 400×). Groups represent, **a**: normal mice + saline; **b**: model mice + saline; **c**: model mice + 5-FU 25 mg/kg; **d**: model mice + 5-FU 25 mg/kg + CMP 50 mg/kg; **e**: model mice + 5-FU 25 mg/kg + CMP 100 mg/kg.

The effect of the combination treatment with CMP and 5-FU on production of ROS, GSH, CAT, SOD, and GSH-Px was evaluated by commercial kits. As shown in Figure 3A, with 5-FU treatment, the level of ROS which may cause oxidative liver injury increased obviously compared to untreated mice. The combination with CMP and 5-FU significantly reduced production of ROS in liver tissues. In contrast, the activities of CAT, SOD and GSH-Px and the level of GSH decreased in livers after 5-FU treatment, which were then significantly reversed by the combination treatment. That is, CMP reduced liver injury caused by 5-FU via suppressing ROS production and increasing levels of CAT, SOD and GSH-Px, and GSH.

We next detected expression of proteins associated with antioxidant using western blotting analysis. The results in Figure 3B,C show that the expression of Nrf-2, HO-1 and GCL was faint in the model and 5-FU group. Fortunately, CMP combined with 5-FU substantially increased the levels of Nrf-2, HO-1 and GCL. Moreover, the increased expression of Nrf-2 induced by the combination treatment were confirmed by immunohistochemistry (Figure 3D). These results indicated that CMP combined with 5-FU effectively reduced liver injury through enhancing the Nrf2 pathway in the livers of mice bearing CT26 xenografts.

### 2.4. Anti-Inflammatory Effect of the Combination with CMP and 5-FU on Liver Tissues

**Figure 4:**
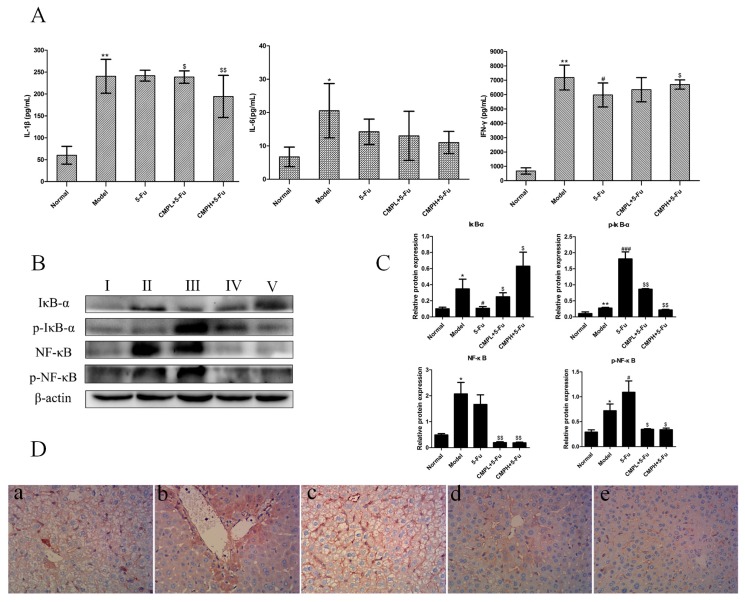
Antiinflammatory effect of the combination treatment with CMP and 5-FU on liver tissues. (**A**) Serum levels of IL-1β, IL-6, IFN-γ. Data are presented as mean ± SD. * *p <* 0.05, ** *p <* 0.01 vs. Normal group; ^#^ *p <* 0.05 vs. Model group; ^$^ *p <* 0.05, ^$$^ *p <* 0.01 vs. 5-FU. (**B**) Western blot analysis of IκBα, p-IκBα, NF-κB, and p-NF-κB in liver tissues. Groups represent, **І**: normal mice + saline; **ІІ**: model mice + saline; **ІІІ**: model mice + 5-FU 25 mg/kg; **ІV**: model mice + 5-FU 25 mg/kg + CMP 50 mg/kg; **V**: model mice + 5-FU 25 mg/kg + CMP 100 mg/kg. (**C**) Semi-quantitative analysis of western blot. Data are presented as mean ± SD. * *p <* 0.05, ** *p <* 0.01 vs. Normal group; ^#^ *p <* 0.05, ^###^ *p <* 0.001 vs. Model group; ^$^ *p <* 0.05, ^$$^ *p <* 0.01 vs. 5-FU group. (**D**) Immunohistochemical staining of NF-κB in liver tissues (magnification 400×). Groups represent, **a**: normal mice + saline; **b**: model mice + saline; **c**: model mice + 5-FU 25 mg/kg; **d**: model mice + 5-FU 25 mg/kg + CMP 50 mg/kg; **e**: model mice + 5-FU 25 mg/kg + CMP 100 mg/kg.

It has been shown that various inflammatory cytokines, such as IL-1β and IL-6, produced during drug-induced liver injury are involved in promoting further liver tissue damage, so we next evaluated serum levels of IL-1β and IL-6. As shown in Figure 4A, CMP combined with 5-FU markedly decreased the level of IL-1β when compared with 5-FU treatment alone, while, that of IL-6 was not changed significantly. Moreover, the level of IFN-γ with strong antitumor activity was obviously decreased in the serum of the 5-Fu group, however, CMPH plus 5-FU significantly upregulated serum levels of IFN-γ, thus improving the antitumor activity of 5-FU.

Next, we detected the effect of the combination treatment on NF-κB pathways. The results of western blotting analysis in Figure 4B,C showed that, expression of NF-κB and p-NF-κB increased significantly in the model and 5-FU group, in comparison with the normal mice without CT26 carcinoma xenografts. After the combination treatment with CMP and 5-FU, the expression of NF-κB and p-NF-κB was significantly reduced. Similarly, phosphorylation of IκB-α, upstream of NF-κB, increased substantially after tumor bearing mice were treated with 5-FU, and CMP combined with 5-FU significantly decreased expression of p-IκB-α in liver tissues. We also confirmed expression of NF-κB with the immunohistochemical method using an antibody against NF-κB, the results of immunohistochemistry were well consistent with the western blot data (Figure 4D), demonstrating downregulation of NF-κB in liver tissues by the combination treatment in comparison with 5-FU treatment alone. These results indicated that CMP combined with 5-FU markedly alleviated inflammation induced by 5-FU via downregulating NF-κB pathway.

### 2.5. Anti-Apoptosis Effect of the Combination with CMP and 5-FU on Liver Tissues

**Figure 5:**
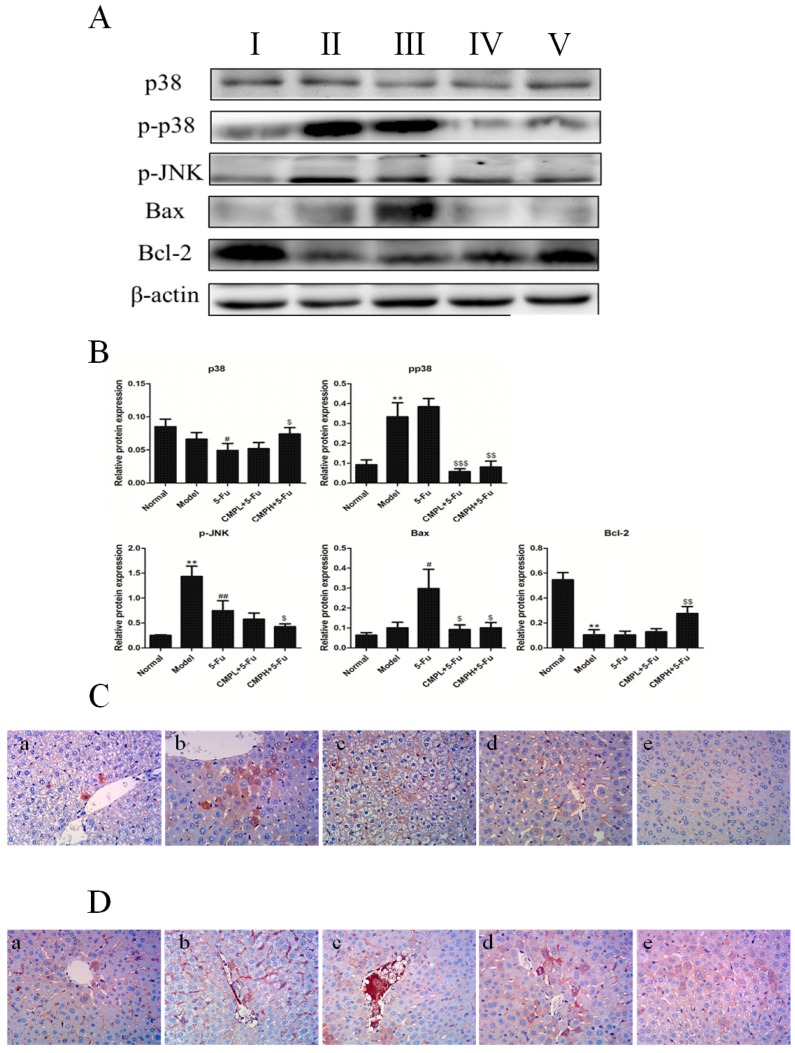
Anti-apoptosis effect of the combination treatment with CMP and 5-FU on liver tissues. (**A**) Western blot analysis of P38, p-P38, p-JNK, Bax, and Bcl-2 in liver tissues. Groups represent, **І**: normal mice + saline; **ІІ**: model mice + saline; **ІІІ**: model mice + 5-FU 25 mg/kg; **ІV**: model mice + 5-FU 25 mg/kg + CMP 50 mg/kg; **V**: model mice + 5-FU 25 mg/kg + CMP 100 mg/kg. (**B**) Semi-quantitative analysis of western blot. Data are presented as mean ± SD. * *p <* 0.05, ** *p <* 0.01 vs. Normal group; ^#^ *p <* 0.05*,* ^##^ *p <* 0.01 vs. Model group; ^$^ *p <* 0.05, ^$$^ *p <* 0.01, ^$$$^ *p <* 0.001 vs. 5-FU group. (**C**,**D**) Immunohistochemical staining of P38 and p-P38 in liver tissues, respectively (magnification 400×). Groups represent, **a**: normal mice + saline; **b**: model mice + saline; **c**: model mice + 5-FU 25 mg/kg; **d**: model mice + 5-FU 25 mg/kg + CMP 50 mg/kg; **e**: model mice + 5-FU 25 mg/kg + CMP 100 mg/kg.

p38 MAPK and c-Jun N-terminal kinase (JNK) play critical roles in 5-FU induced liver injury, thereby we further detected expression of p-p38 and p-JNK by western blotting analysis. As shown in Figure 5A,B, phosphorylation of p38 and JNK significantly increased in the model group and 5-FU group when compared with normal mice without CT26 carcinoma xenografts. However, CMP combined with 5-FU obviously decreased the phosphorylation levels of p38 and JNK. Additionally, expression of the pro-apoptotic protein Bax in liver tissues was obviously increased and that of the anti-apoptotic protein Bcl-2 was significantly decreased by 5-FU treatment, which indicated apoptosis in the liver tissues. The combination treatment with CMP and 5-FUsignificantly attenuated increase of Bax and decrease of Bcl-2, thereby inhibiting liver injury induced by hepatocyte apoptosis.

To further confirm the effect of the combination treatment on proteins related to apoptosis, we detected the expression of associated proteins in liver tissues by immunohistochemistry. As shown in Figure 5C,D, expression of p38 and p-p38 increased significantly in liver tissues of 5-FU treated mice, however, CMP combined with 5-FU significantly attenuated the expression of p38 and p-p38, comfirming the antiapoptosis of the combination treatment.

## 3. Discussion

5-FU, an effective and clinical commonly used chemotherapeutic agent, can significantly inhibit the proliferation of tumors, at the cost of reducing the body mass, inducing liver injury, and suppressing the immunological function [8]. Previous studies have demonstrated liver injury induced by 5-FU in animal models, and most recently, pathogenetic mechanisms of liver injury induced by 5-FU has been investigated in experimental models [23,24]. In the present study, we also verified liver injury induced by 5-FU in CT26-bearing mice and found that CMP was hepatoprotective against 5-FU-induced liver injury as evidenced by serum biochemical parameters, including AST and ALT, as well as by morphological and histological assessments. Furthermore, CMP could improve the immunosuppressive effect and the abnormal situation on the organs induced by 5-FU, thereby enhancing its anti-tumor action and alleviating liver injury. The mechanism may be correlated with regulation of NF-κB, Nrf2-ARE and MAPK/P38/JNK pathways.

Liver injury could alter the permeability of the membrane, thus leading to increased release of some hepatospecific enzymes, including aspartate aminotransferase (AST) and alanine aminotransferase (ALT) [25]. It has been extensively reported that oxidative stress is a key element promoting production of free radicals, and the excessive free radicals could, therefore, induce damage, destruction of various tissues and further oxidative injury [26,27,28,29]. In this regards, free radicals including ROS are found to be responsible for 5-FU-induced liver injury, and, thus, inhibition of ROS in liver tissues may be an effective and attractive strategy to reduce liver injury induced by 5-FU treatment. In this study, CMP substantially reduced release of AST and ALT and effectively alleviated liver damage caused by 5-FU as evidenced by HE analysis, revealing hepatoprotective effect of CMP in 5-FU treated CT26-bearing mice.

A major mechanism of the cellular defense against oxidative stress is the activation of NF-E2-related nuclear factor 2 (Nrf2)-antioxidant response element (ARE) signaling, which acts as a central controller of many detoxification and antioxidant enzymes that are responsible for diverse cytoprotective processes [30]. Most recently, the activation of the NRF2/ARE pathway has been identified as a protective response that protects against liver injury [31,32]. Under normal conditions, Nrf2 is localized in the cytoplasm by interaction with Kelch-like ECH associating protein 1 (Keap1), a master regulator preventing nuclear translocation of Nrf2 and inducing Nrf2 ubiquitin-mediated degradation [33,34]. Upon exposure to ROS or other stimuli, Keap1 is degraded, and Nrf2 is subsequently activated and translocates into the nucleus where it binds to the antioxidant response element (ARE), a regulatory enhancer region within gene promoters, thus enhancing production of many phase II detoxifying and antioxidant enzyme genes such as glutathione peroxidase (GPx), glutathione-S-transferase (GST), heme oxygenase (HO), glutamate cysteine ligase (GCLc) and superoxide dismutase (SOD), that protects cells from oxidative damage [35,36,37]. In the present study, our results showed that CMP combined with 5-FU enhanced nuclear translocation of Nrf2, decreased levels of Keap1, and elevated expression of HO-1 and GCL compared with 5-FU treatment alone, indicating upregulation of the Nrf2-ARE pathway in CMP-mediated hepatoprotective effect. Moreover, decreased levels of T-SOD, CAT, GSH-Px, and GSH, which play unequivocal roles in detoxifying the reactive toxic metabolites of many toxins, were substantially restored by the combined treatment, confirming the antioxidant property of CMP in 5-FU treated CT26 xenografts.

Uncontrolled inflammation is also generally appreciated as an aggravating factor for drug-related tissue injury [38,39]. Nuclear factor kappa-B (NF-κB), is a key transcription factor and master regulator of inflammatory processes, which activates genesparticularly involved in the inlammatory response, such as proinflammatory cytokines IL-1β, and IL-6 [40]. A tight regulation of NF-κB signaling is essential to maintain a beneficial level of inflammatory cytokines and chemokines. Under normal circumstances, NF-κB is located in the cytoplasm in an inactive form by binding to inhibitory factor kappaB-alpha(IκBα) which is controlled by the upstream of the IκB kinase (IKK) complex [41]. In response to a variety of stimuli, NF-κB dissociates from IκB-α and then translocates into the nuclear to modulate the transcription of downstream genes including chemokines, cytokines, and anti-apoptotic Bcl-2 family members [42,43]. Our results suggested that CMP in combination with 5-FU could significantly decrease levels of inflammatory cytokines including IL-1β and IL-6 and markedly down-regulate the NF-κB pathway, possibly contributing to the hepatoprotective effect of CMP in 5-FU treated CT26-bearing mice.

Additionally, liver injury are believed to be associatedwith hepatocyte apoptosis [44,45] which is modulated by mitogen activated protein kinases (MAPKs), including p38 MAPK and c-Jun N-terminal kinase (JNK) which transduce extracellular signals to various subcellular compartments regulating a variety of biologic processes, e.g. cell survival and apoptosis, inflammation, and necrosis [46,47,48]. Persistent activation of p38 MAPK and JNK is proposed to be related to hepatocyte apoptosis [49,50]. Bcl-2 family members, including pro-apoptotic protein Bax and anti-apoptotic protein Bcl-2, have also been extensively studied as regulators of apoptosis [51]. The results of the present study showed that CMP could obviously decrease the expression of pro-apoptotic proteins pp38 and Bax, increase levels of the anti-apoptotic protein Bcl-2, thus alleviating apoptosis of the hepatic cells.

In summary, this study demonstrated that CMP in combination with 5-FU could potentiate antitumor action, reduce toxicity and prevent liver injury via inhibiting inflammation and apoptosis, and enhancing antioxidant in liver tissues, compared with 5-FU treatment alone. Further study revealed that CMP in combination with 5-FU enhanced regulation of NF-κB, p38 MAPK, Bcl-2 protein family and Nrf2-ARE signaling pathways, which is, to some extent, responsible for alleviated toxicity to liver tissue. Collectively, our data provides a scientific rationale that CMP in combination with 5-FU exhibited enhanced antitumor efficacies with alleviated side effects and toxicity, highlighting its potential in colorectal cancer treatment.

## 4. Materials and Methods

### 4.1. Chemicals and Reagents

Carboxymethyl Pachymaran was a gift from Hunan Butian Pharmaceutical Co., Ltd. (Huaihua, China)country; Fluorouracil Injection was provided by Shanghai XudongHaipu Pharmaceutical Co., Ltd. (Shanghai, China); alanine aminotransferase (ALT) assay kitand aspartate aminotransferase (AST) assay kit were purchased from Beijing Leadman Biochemistry Co., Ltd. (Beijing, China); Superoxide dismutase (SOD), catalase (CAT), glutathione (GSH), glutathione and catalase (GSH-Px), and reactive oxygen species (ROS) kits were purchased from Nanjing Jiancheng Bioengineering Institute (Nanjing, China); ELISA kits specific for interleukin -1β (IL-1β), interleukin-6 (IL-6) and interferon-γ (IFN-γ) were purchased from the Huamei Institute of Biotechnology (Wuhan, China); β-actin, NF-κB, p-NF-κB, IκB-α, p-IκB-α, p-p38, p-JNK, GCL, Bax, Bcl-2, Keap1, Nrf2, p38, and HO-1 were purchased from Santa Cruz Biotechnology (Santa Cruz, CA, USA). All secondary antibodies were purchased from Cowin Biotech (Beijing, China).

### 4.2. Animals and Cells

Male Balb/c mice, weighing18–20 g, were purchased from Vital River Laboratory Animal Technology Co., Ltd. (Beijing, China) and were housed for 3 d prior to the experiments. Mice, with free access to food and water, were kept on a 12 h light/dark cycle with controlled humidity (50%–70%) and temperature (20–24 °C). All animal care and experimental protocols used in this study were approved by the Institutional Animal Care and Use Committee at the Institute of Medicinal Plant Development, Chinese Academy of Medical Sciences (No. SLXD-15-10-15).Mouse colon cancerCT26 (CT26.WT) cells were purchased from the Chinese Academy of Medical Sciences Basic Medicine Cell Center (Beijing, China). Cells were cultured in RPMI-1640 medium containing 10% fetal bovine serum (FBS), 100 U/mL penicillin, and 100 µg/mL streptomycin at 37 °C in an atmosphere of 5% CO_2_.

### 4.3. CT26 Xenografts Model

A total of 6 × 10^7^ of CT26 cells were inoculated subcutaneously into the right flanks of mice. 24 h after CT26 cells inoculation, mice were randomly divided into four groups of ten mice each: model group, 5-FU (25 mg/kg) group, CMP (50 mg/kg, CMPL) + 5-FU (25 mg/kg) group, CMP (100 mg/kg, CMPH) + 5-FU (25 mg/kg) groups. Another ten mice without CT26 cells inoculation was set as the normal group. Mice in normal group and model group were orally administered with distilled water (20 mL/kg, 1 time/day) and were intraperitoneally injected (ip.) with normal saline (10 mL/kg, 1 time/2 days). Mice in 5-FU group were intraperitoneally injected with 5-FU (25 mg/kg, 1 time/2 days), Mice in combined therapy groups were orally administrated with CMP (50 or 100 mg/kg, 1 time/day) and intraperitoneally injected with 5-FU (25 mg/kg, 1 time/2 days). Each group was administered continuously for 14 days.

### 4.4. Organ Index and Tumor Inhibition Rate

After the last administration, mice were weighted and sacrificed. Tumor, spleen, liver and kidney were isolated and weighted to calculate tumor inhibition rates and organ indexes. Tumor inhibition rate = (the average tumor weight of model group − the average tumor weight of treated group)/the average tumor weight of model group × 100%, organ index = organs weight (mg)/body weight (g).

### 4.5. Peripheral White Blood Cells (WBCs) and Bone Marrow Nucleated Cells (BMNC) Number

Blood (10 μL) taken from tail tip was mixed with 3% acetic acid (190 μL). The number of WBCs were counted under microscope. In addition, marrow taken from the right femurs was also mixed with 3% acetic acid, after which the BMNCs were counted using blood count plates.

### 4.6. Determination of ALT and AST Activities in Serum

ALT and AST activities in serum were tested using an automatic biochemical analyzer, according to the manufacturer's instructions.

### 4.7. Determination of Lipid Peroxidation in Liver Tissues

The liver tissues were isolated and homogenized with ice-cold saline (1:9 (*w*/*v*)) using a homogenizer. After centrifuged at 3000 rpm/min at 4 °C for 15 min, the supernatant was collected and used for assay of hepatic components and enzymes. Levels of ROS, GSHs, CAT, SOD and GSH-Px were detected using commercial kits following the manufacturer's instructions.

### 4.8. Histopathological Analysis

Liver tissues were fixed in 10% formalin solution, dehydrated and embedded in paraffin. Then the paraffin embedded samples were sectioned at 5 μm. For histological analysis, sections were stained with hematoxylin-eosin (H&E) using standard techniques, and were observed under a microscope at the magnification of 400 × and were then photographed. Histological damage was calculated using the following score system (LDI score): 0, absent (no destruction); 0.5, few (0%–20% destruction); 1, mild (20%–50% destruction); 2, moderate (50%–80% destruction); 3, severe (80%–100% destruction).

### 4.9. Immunohistochemical (IHC) Analysis of Liver Tissue

The liver tissues were fixed in formalin solution, dehydrated with gradually increasing concentrations of ethanol, embedded in paraffin, and sectioned. The 5 μm sections were blocked with a buffered blocking solution (3% bovine serum albumin in phosphate-buffered saline (PBS) for 15 min. Then, the sections were co-incubated with primary antibody for NF-kB, p38, p-p38 and Nrf2 at a dilution of 1:50 in PBS (*v*/*v*), at 4 °C overnight, followed by washing with PBS and co-incubating with secondary antibody at a dilution of 1:500 in PBS (*v*/*v*), at room temperature for 1 h. Thereafter, sections were washed with Tris-HCl 0.05 M, pH 7.66, and then co-incubated with a 3, 3′-diaminobenzidine solution in darkness, at room temperature for 10 min. The sections were washed with Tris-HCl, stained with hematoxylin according to standard protocols, and observed under a light microscope. The Image-ProPlus 4.5 Software (Media Cybernetics, Rockville, MD, USA) was used to analyze the expression of proteins.

### 4.10. Western Blotting Analysis

Briefly, liver tissues were homogenized in a standard RIPA buffer supplemented with a cocktail of protease and phosphatase inhibitors. The homogenate was then centrifuged at 15,000× *g* at 4 °C for 10 min. The protein concentration was determined using a BCA Protein Assay Kit (Beyotime Institute of Biotechnology, Shanghai, China). The proteins were separated on SDS-PAGE, and transferred to polyvinylidene fluoride membrane (Millipore, Boston, MA, USA), which were then blocked in 5% skim milk for 2 h at room temperature. Membranes were incubated with appropriate concentration of primary antibodies at 4 °C overnight, and were then washed three times with TBST, followed by incubated at room temperature for 2 h with horseradish peroxidase (HRP) conjugated secondary antibodies. Finally, Protein expression was measured using an enhanced chemistry luminescence (ECL) method. The images were captured by a Bio-Spectrum gel imaging system.

### 4.11. Detection of IL-1β, IL-6 and IFN-γ in Serum

Cytokines IL-1β, IL-6 and IFN-γ in the serum were measured using mouse ELISA kits specific for each cytokine according to the manufacturer’s instructions.

### 4.12. Statistical Analysis

Data were expressed as Mean ± SD for ten animals in each group and statistically evaluated with SPSS 17.0 software (IBM Corporation, New York, NY, USA). Differences between each group were analyzed by one-way (ANOVA) and Tukey’s Post Hoc test (TTEST). *p* < 0.05 was considered statistical significance.
